# A deep learning‐based interpretable decision tool for predicting high risk of chemotherapy‐induced nausea and vomiting in cancer patients prescribed highly emetogenic chemotherapy

**DOI:** 10.1002/cam4.6428

**Published:** 2023-08-23

**Authors:** Jingyue Zhang, Xudong Cui, Chong Yang, Diansheng Zhong, Yinjuan Sun, Xiaoxiong Yue, Gaoshuang Lan, Linlin Zhang, Liangfu Lu, Hengjie Yuan

**Affiliations:** ^1^ Department of Pharmacy Tianjin Medical University General Hospital Tianjin China; ^2^ School of Mathematics Tianjin University Tianjin China; ^3^ Department of Pharmacy Tianjin Huanhu Hospital Tianjin China; ^4^ Department of Medical Oncology Tianjin Medical University General Hospital Tianjin China; ^5^ Academy of Medical Engineering and Translational Medicine Tianjin University Tianjin China

**Keywords:** chemotherapy‐induced nausea and vomiting, deep forest, highly emetogenic chemotherapy, kidney function, predictive model, risk factors

## Abstract

**Objective:**

This study aims to develop a risk prediction model for chemotherapy‐induced nausea and vomiting (CINV) in cancer patients receiving highly emetogenic chemotherapy (HEC) and identify the variables that have the most significant impact on prediction.

**Methods:**

Data from Tianjin Medical University General Hospital were collected and subjected to stepwise data preprocessing. Deep learning algorithms, including deep forest, and typical machine learning algorithms such as support vector machine (SVM), categorical boosting (CatBoost), random forest, decision tree, and neural network were used to develop the prediction model. After training the model and conducting hyperparameter optimization (HPO) through cross‐validation in the training set, the performance was evaluated using the test set. Shapley additive explanations (SHAP), partial dependence plot (PDP), and Local Interpretable Model‐Agnostic Explanations (LIME) techniques were employed to explain the optimal model. Model performance was assessed using AUC, F1 score, accuracy, specificity, sensitivity, and Brier score.

**Results:**

The deep forest model exhibited good discrimination, outperforming typical machine learning models, with an AUC of 0.850 (95%CI, 0.780–0.919), an F1 score of 0.757, an accuracy of 0.852, a specificity of 0.863, a sensitivity of 0.784, and a Brier score of 0.082. The top five important features in the model were creatinine clearance (Ccr), age, gender, anticipatory nausea and vomiting, and antiemetic regimen. Among these, Ccr had the most significant predictive value. The risk of CINV decreased with increased Ccr and age, while it was higher in the presence of anticipatory nausea and vomiting, female gender, and non‐standard antiemetic regimen.

**Conclusion:**

The deep forest model demonstrated good discrimination in predicting the risk of CINV in cancer patients prescribed HEC. Kidney function, as represented by Ccr, played a crucial role in the model's prediction. The clinical application of this predictive tool can help assess individual risks and improve patient care by proactively optimizing the use of antiemetics in cancer patients receiving HEC.

## 1 INTRODUCTION

Chemotherapy‐induced nausea and vomiting (CINV) is the most common side effect that not only impacts the quality of life but also affects treatment outcomes.^1^, ^2^ Chemotherapy drugs are classified into different risk grades based on the likelihood of vomiting without preventive treatment, namely high, moderate, low, and minimal risk, corresponding to vomiting incidences >90%, 30%–90%, 10%–30%, and < 10%, respectively. Despite advancements in antiemetic therapies, some cancer patients, particularly those receiving highly emetogenic chemotherapy (HEC), still experience CINV. The latest NCCN guidelines recommend a 3‐drug regimen based on neurokinin‐1 receptor antagonist (NK‐1 RA) consisting of NK‐1 RA, 5‐hydroxytryptamine‐3 receptor antagonist (5‐HT3 RA), and dexamethasone, or a 3‐drug regimen based on olanzapine consisting of olanzapine, 5‐HT3 RA, and dexamethasone, or a 4‐drug regimen including olanzapine, NK‐1 RA, 5‐HT3 RA, and dexamethasone for HEC patients.^3^ However, even with these recommended antiemetics, HEC can still cause CINV in 30% of patients.^4^, ^5^ Hence, it is crucial to develop a tool for identifying CINV risk, which can guide the implementation of more effective antiemetic prevention strategies for cancer patients receiving HEC.

Several patient‐specific factors are associated with the risk of CINV development. Previous meta‐analyses have identified various risk factors, including patient expectations of CINV following chemotherapy, younger age, female gender, history of morning sickness during pregnancy, chemotherapy cycle, and inadequate sleep (<7 h) before chemotherapy, among others.^6^, ^7^, ^8^ These factors have been considered in guidelines such as NCCN, ESMO, and MASSC, which recommend selecting a regimen based on the drug with the highest vomiting risk and patient‐related risk factors.^9^, ^10^ However, previous studies have overlooked factors affecting drug metabolism. The drug concentration in a patient's body may influence the occurrence of CINV. Nonetheless, there is limited research investigating the impact of liver and kidney function on CINV. This study is the first to incorporate liver and kidney function as indicators in studies related to CINV risk factors.

In recent years, artificial intelligence techniques have been widely employed in the medical field,^11^ including medical diagnosis,^12^, ^13^ medical imaging,^14^, ^15^, ^16^ and diagnostic prediction.^17^, ^18^ To incorporate patient‐specific risk factors into the choice of antiemetic treatment, we developed a predictive model based on deep learning, capable of estimating individual CINV risk within a 0 to 14‐day window. Compared to traditional machine learning, deep learning can identify better features during model training, and it does not require complex classification functions as long as the extracted features are sufficiently informative.^19^ Zhou et al.^20^ proposed the deep forest method by combining deep learning with random forest. The deep forest can automatically determine the model complexity based on the data size and maintain stable and effective learning performance even with limited training data. However, deep learning is considered a “black box” approach, making it challenging to interpret the decision process. Therefore, the application of deep learning in medical prediction should be approached cautiously. To address this issue, the Shapley additive explanations (SHAP), the Partial Dependence Plot (PDP), and the Local Interpretable Model‐Agnostic Explanations (LIME) techniques have been designed to provide clinicians with visualizations that help understand the relationship between each feature and the outcome.^21^, ^22^, ^23^ Hence, the primary objective of this study was to develop a deep forest model as an interpretable decision tool for individually predicting the occurrence of CINV in patients received HEC.

## 2 METHODS

### 2.1 Study population and data

This retrospective observational study included cancer patients who were prescribed HEC and were enrolled in the Department of Oncology at Tianjin Medical University General Hospital, China, from January 2018 to December 2021. The study had specific inclusion and exclusion criteria. The inclusion criteria were as follows: (1) age over 18 years, (2) diagnosis of cancer, (3) patients who received HEC, and (4) non‐use of self‐prescribed treatments or supplements. The exclusion criteria were: (1) pregnancy or lactation, (2) current radiotherapy, (3) presence of other diseases that may cause nausea and vomiting, and (4) chronic use of CYP3A4 inducers for less than 4 weeks or inhibitors for less than 1 week prior to chemotherapy.^24^ The ethics committees of Tianjin Medical University General Hospital approved this study (No. IRB2021‐WZ078).

The primary endpoint of the study was the complete response (CR), which was defined as the absence of vomiting and the absence of rescue therapy within 0–14 days from the start of chemotherapy. To collect prospective CINV outcome data, a standardized electronic patient diary was utilized. At the beginning of each study, patients were provided with a daily electronic diary to record the number of vomiting episodes, the number and intensity of nausea, and the use of rescue therapy within 0–14 days from the start of chemotherapy. In cases where patients were discharged from the hospital, they were contacted via telephone or instant messaging to ensure the accurate completion of the electronic diary. The nurse staff recorded the CINV data daily from the electronic diary.

Data collection involved gathering patient demographics, liver and kidney function indicators, and potential predictors of CINV. These included gender, age, highly emetogenic drug (HED),^3^, ^25^ antiemetic regimen, history of drinking, creatinine clearance (Ccr), alanine transaminase (ALT), aspartate transaminase (AST), alkaline phosphatase (ALP), total proteins (TP), globulin (GLB), albumin (ALB), total bilirubin (TBILI), direct bilirubin (DBILI), history of morning sickness during a prior pregnancy (if applicable), anticipatory nausea and vomiting, use of non‐prescribed antiemetics at home, chemotherapy cycle, nausea or vomiting in the previous cycle, and sleeping time on the night before chemotherapy. The creatinine clearance (Ccr) was calculated using the Cockcroft‐Gault formula:

$$\begin{array}{c} C\mathrm{cr}\left(\mathrm{mL}/\min\right)=\frac{\left.\left(140-\mathrm{age}\right)\times \text{Lean Body Weight}(\mathrm{kg}\right)}{\text{Serum Creatinine}\;\left(\mathrm{mg}/\mathrm{dL}\right)\times 72}\\ \;\times \left(0.85\;\text{if female}\right) \end{array}$$

### 2.2 Model development

**FIGURE 1:**
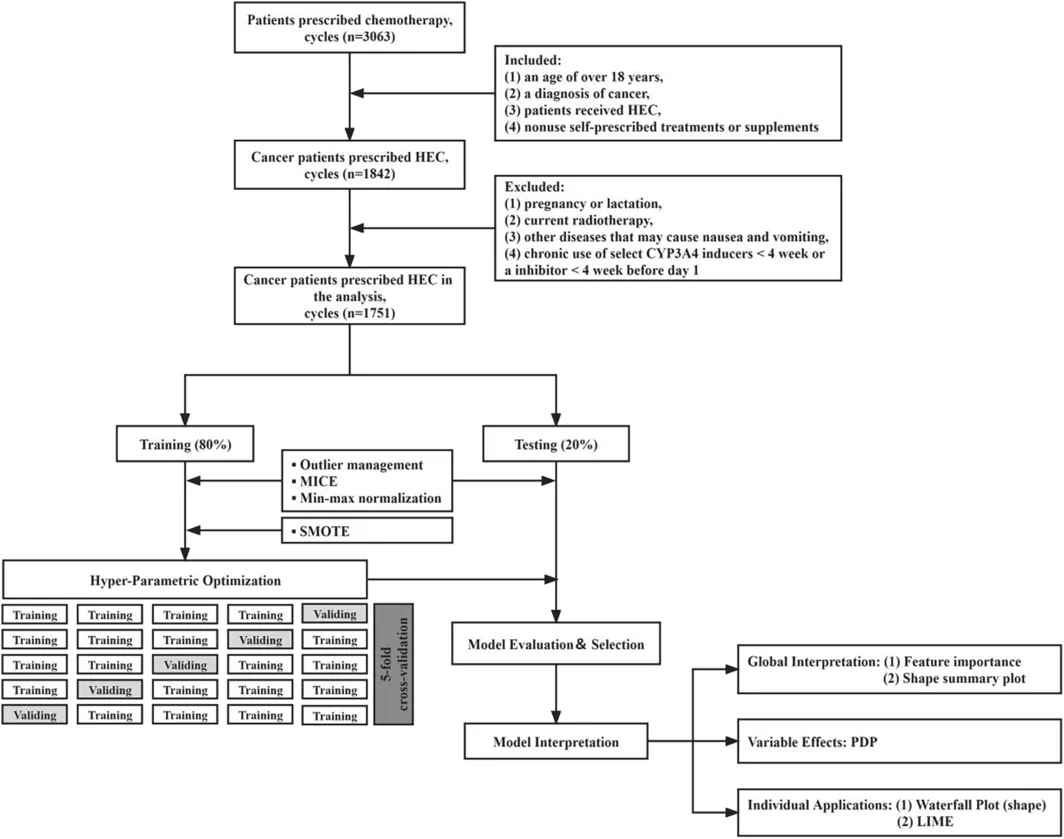
Screening process of patients and the main flow of the study. MICE, Multivariate Imputation Chained Equations; PDP, Partial Dependence Plot; LIME, Local Interpretable Model‐Agnostic Explanations; SMOTE, Synthetic Minority Oversampling Technique.

The data were divided into a training set (80%) and a test set (20%) using a stratified random permutations method (Figure 1). The training set was used to train and optimize the model, while the test set was used to evaluate the true predictive power of the model.

To address the impact of outliers on the model's performance, variables with outliers were transformed into logarithmic space to mitigate their effects. For missing data, variables with missing values exceeding 40% were removed to minimize potential bias. The remaining variables were imputed using the Multivariate Imputation Chained Equations (MICE) method.^26^ Details of the missing value information for each variable are provided in Table S1. Given that the variables included in the analysis had different dimensions and orders of magnitude, it was important to normalize them to avoid bias towards higher‐valued variables and weaken the role of lower‐valued variables, which could result in low reliability. Min‐max normalization was applied, which linearly transformed the original data into the [0, 1] interval. The training set had an imbalance between the CR group and the non‐CR group, with a ratio of approximately 6:1. To address this imbalance and avoid bias towards the CR group, the Synthetic Minority Oversampling Technique (SMOTE)^27^, ^28^ was employed in the training set. The relationship between each variable was examined using Kendall correlation analysis.

Deep forest^20^ was selected to develop the prediction model. A 20‐dimensional sample was firstly input to multi‐grained scanning, which was scanned by 5‐dim, 10‐dim and 15‐dim windows to obtain 16 5‐dimensional vectors, 11 10‐dimensional vectors, and 6 15‐dimensional vectors, which were input to completely random forest and random forest, and the output results were aggregated into 66‐dimensional vectors. The 66‐dimensional vectors were input to the cascade forest, and the 66‐dimensional vectors were aggregated with the outputs of the first and second layers of the cascade forest, and the results of the third layer were averaged, and then the maximum value was taken to produce the prediction results (Figure S1). Five typical machine learning models, namely support vector machine (SVM), categorical boosting (CatBoost), random forest, decision tree, and neural network, were chosen as comparators. Each model underwent 5‐fold cross‐validation and hyperparameter optimization (HPO) using the training set. The performance of the models was compared with an independent test set. HPO was conducted using the Optuna python open‐source optimization framework (version 2.10.0). The performance of the models was evaluated using metrics such as the area under the receiver operating characteristic curve (AUC), accuracy, specificity, sensitivity, and F1 score. Calibration plots were generated to assess the consistency of the model's predicted probabilities with the actual probabilities, and Brier scores were used to evaluate the calibration of the model. To interpret the model at both global and local levels and avoid relying solely on a single interpretation algorithm, SHAP, PDP, and LIME techniques were applied. All statistical analyses were performed using Python (version 3.8.3) and SPSS (version 25.0).

## 3 RESULTS

### 3.1 Clinical characteristics

A total of 756 patients who underwent 1751 chemotherapy cycles (median 3; range 1–14) were included in this study. The median age of the patients was 64 years, with 38.6% being female and 65.1% diagnosed with lung cancer. Among the patients, 13.6% had a history of morning sickness during a prior pregnancy, and 27.4% had a history of alcohol consumption (Table 1). Additionally, 29.9% of the patients slept less than 7 h before chemotherapy, and 21.8% expected to experience CINV after treatment. Carboplatin AUC≥4 or μmol/L cisplatin‐based chemotherapy was administered in 49.3% and 45.1% of patients, respectively (Table 1). As for antiemetic prophylaxis, 80.5% of patients received 5‐HT3 RA, NK‐1 RA, and dexamethasone before each chemotherapy treatment, while 19.5% received 5‐HT3 RA and dexamethasone only. Detailed liver and kidney function data are presented in Table 1.

**TABLE 1:** Patient and treatment characteristics.

Patient characteristic	Patients (*n* = 756)
Median patient age	64 (19–85)
Gender, *n* (%)	
Male	464 (61.4%)
Female	292 (38.6%)
Type of cancer, *n* (%)	
Lung	492 (65.1%)
Breast	33 (4.4%)
Gynecologic	48 (6.3%)
Gastrointestinal	23 (3.0%)
Esophageal	27 (3.6%)
Others	132 (17.5%)
Missing	1 (0.1%)
History of morning sickness during a pregnancy, *n* (%)	
Yes	103 (13.6%)
No	189 (25.0%)
Inapplicable	464 (61.4%)
History of drinking, *n* (%)	
Yes	207 (27.4%)
No	251 (33.2%)
Missing	298 (39.4%)

Patient/treatment characteristic	Cycles (*n* = 1751)
Median number of cycles (range)	3 (1–14)
Sleep <7 h before chemotherapy, *n* (%)	
Yes	524 (29.9%)
No	1227 (70.1%)
Anticipatory nausea and vomiting, *n* (%)	
Yes	382 (21.8%)
No	1369 (78.2%)
Type of chemotherapy, *n* (%)	
Cisplatin based	864 (49.3%)
Carboplatin AUC≥4 based	790 (45.1%)
AC based	97 (5.6%)
Antiemetic therapy, *n* (%)	
5HT3 RA + NK‐1 RA + dexamethasone	1409 (80.5%)
5HT3 RA + dexamethasone.	342 (19.5%)
Ccr(mL/min), mean (SD)	110.4 (40.5)
TP(g/L), mean (SD)	69.7 (6.8)
ALB(g/L), mean (SD)	38.2 (5.1)
GLB(g/L), mean (SD)	31.5 (5.4)
AST(U/L), mean (SD)	23.0 (11.9)
ALT(U/L), mean (SD)	21.8 (15.1)
TBILI(μmol/L), mean (SD)	8.8 (3.6)
DBILI(μmol/L), mean (SD)	2.5 (1.4)
ALP(U/L), mean (SD)	103.5 (187.0)

Abbreviations: AC, Anthracycline and cyclophosphamide; ALB, albumin; ALP, alkaline phosphatase; ALT, alanine transaminase; AST, aspartate transaminase; Ccr, creatinine clearance; DBILI, direct bilirubin; GLB, globulin; NK‐1 RA, neurokinin‐1 receptor antagonist; TBILI, total bilirubin; TP, total proteins; 5‐HT3 RA, 5‐hydroxytryptamine‐3 receptor antagonist.

### 3.2 CINV clinical outcomes

**FIGURE 2:**
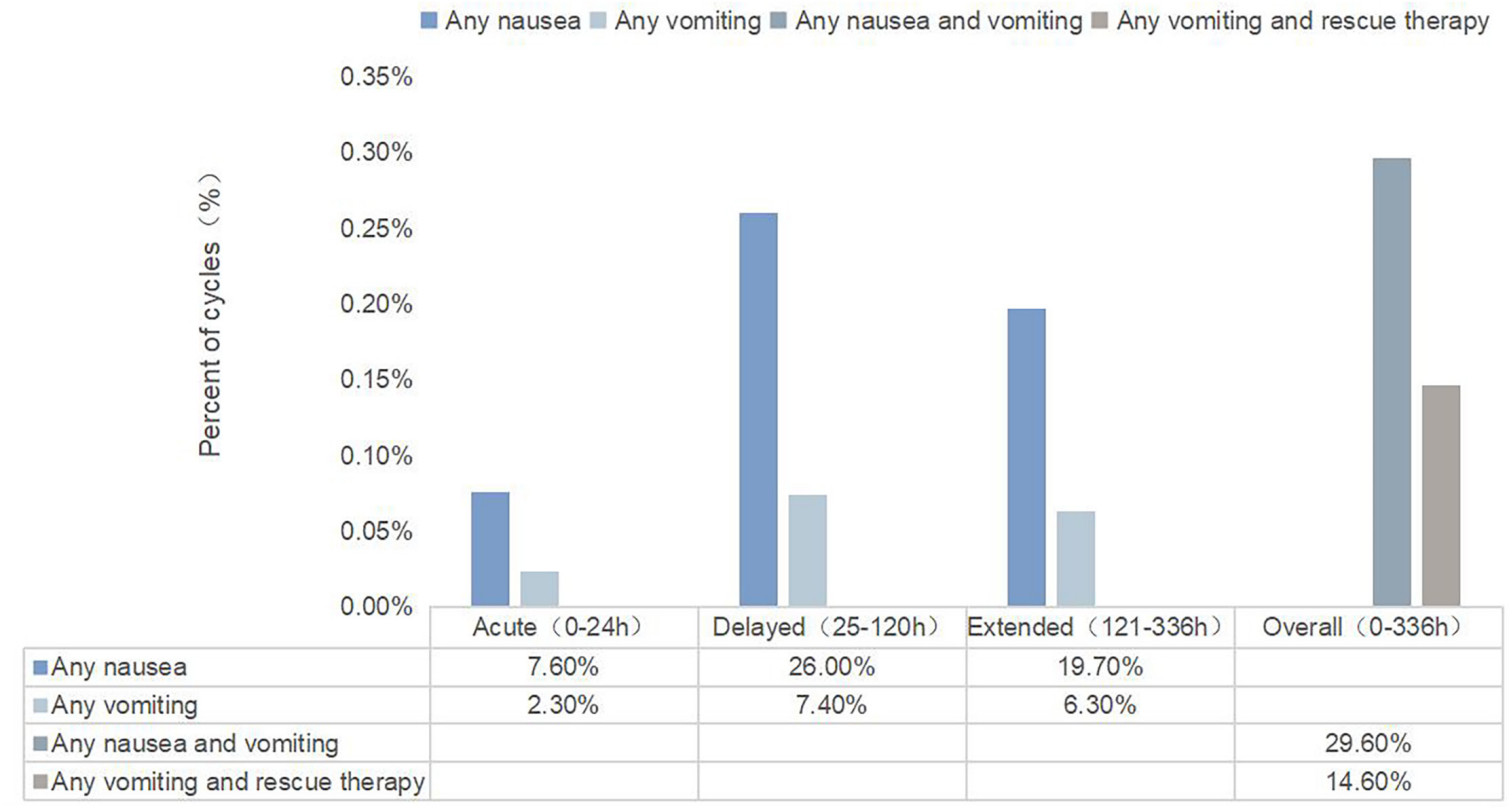
CINV outcomes data.

During the 1751 chemotherapy cycles, 2.3% of cycles reported vomiting and 7.6% reported any nausea within the first 24 h after initiating chemotherapy. From days 2 to 5, 26.0% of cycles experienced delayed nausea, while 7.4% experienced delayed vomiting. From days 6 to 14, the proportions of cycles with extended nausea and vomiting were 19.7% and 6.3%, respectively. When data from days 0 to 14 were combined, any nausea or vomiting occurred in 29.6% of cycles, and complete response (CR) was not observed in 14.6% of cycles (Figure 2).

### 3.3 Construction and evaluation of model

**FIGURE 3:**
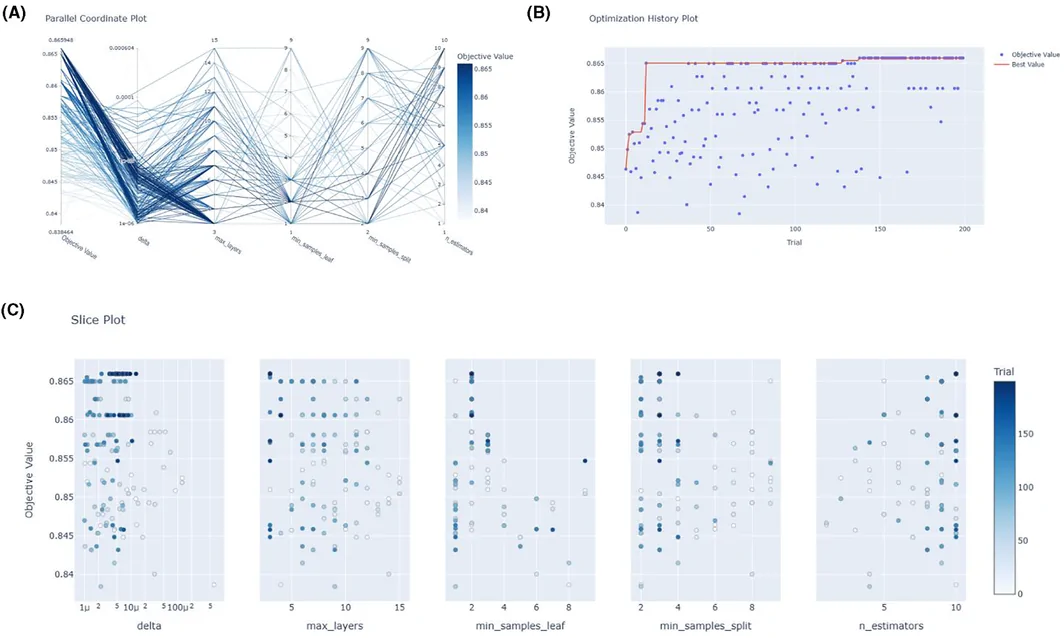
HPO process for the deep forest model. (A) parallel coordinate system plot showing the distribution of hyperparameters in the deep forest model during HPO, where dark colors are matched with higher AUC values; (B) optimization history plot showing the trajectory of the evolution of the optimal values with the number of training sessions during the HPO process; (C) slice plot shows the correlation between each hyperparameter and AUC.

Six models underwent hyperparameter tuning using HPO in the training set, and the hyperparameter combination with the highest AUC score was selected after 200 trials. The deep forest model was ultimately trained with the following hyperparameter settings: n_estimators = 10, max_layers = 3, min_samples_split = 3, delta = 3.508e‐06, min_samples_leaf = 2 (Figure 3). The hyperparameters for the final settings of the other models are shown in Table S2.

**FIGURE 4:**
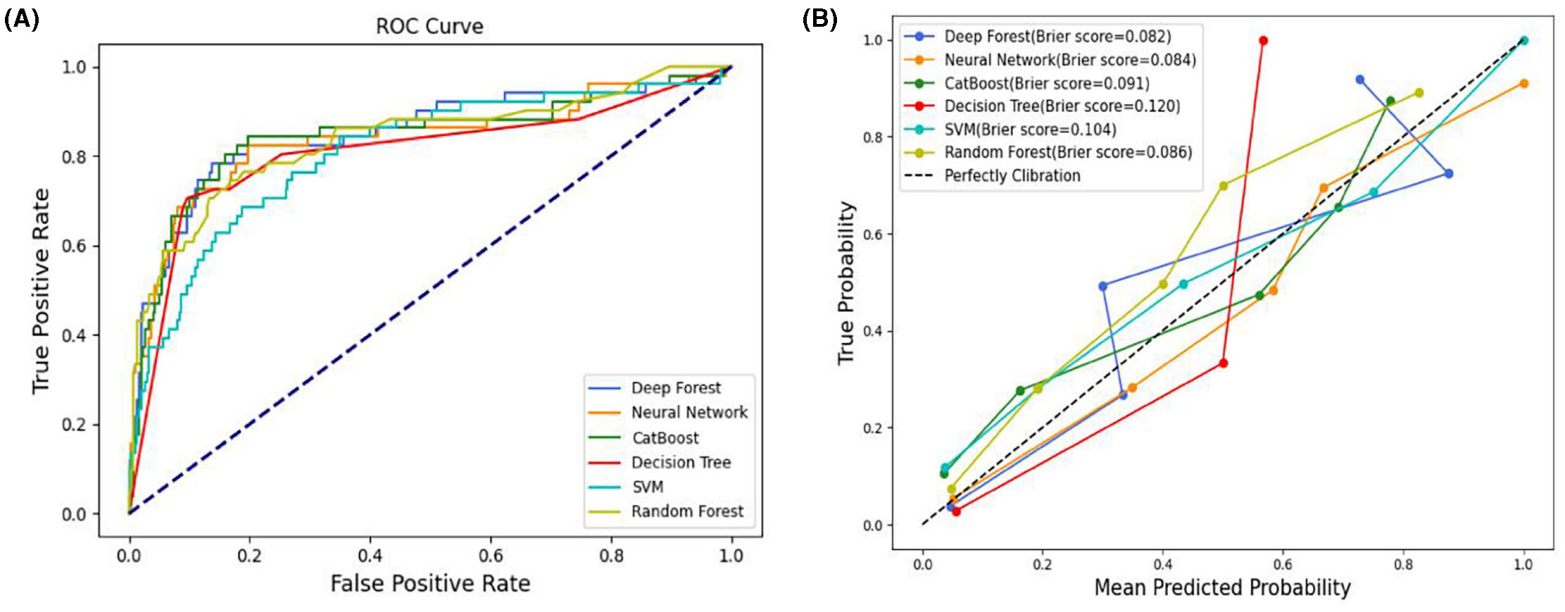
Performance of the models. (A) ROC curves of the six models; (B) calibration curves of the six models.

All samples in the test set were assigned risk outcomes based on the trained models. Model performance was assessed using AUC, F1 score, accuracy, specificity, and sensitivity. The trained deep forest model achieved an AUC of 0.850 (95%CI, 0.780–0.919), an accuracy of 0.852, a specificity of 0.863, a sensitivity of 0.784, and an F1 score of 0.757. It demonstrated good discrimination and outperformed typical machine learning models, including neural network, CatBoost, decision tree, SVM, and random forest (Table 2, Figure 4A). In terms of calibration, the deep forest model also exhibited the best performance compared to other models, as indicated by the Brier score of 0.082 (Figure 4B).

**TABLE 2:** Computational performance of deep forest as compared to other models.

Model	AUC(95%)	Accuracy	Specificity	Sensitivity	F1	Brier
Random forest	0.837 (0.766–0.908)	0.818	0.830	0.745	0.714	0.086
SVM	0.807 (0.736–0.879)	0.795	0.813	0.686	0.682	0.104
Catboost	0.843 (0.770–0.917)	0.809	0.803	0.843	0.720	0.091
Neural network	0.836 (0.783–0.916)	0.806	0.803	0.824	0.715	0.084
Decision tree	0.807 (0.728–0.886)	0.875	0.903	0.706	0.773	0.120
Deep forest	0.850 (0.780–0.919)	0.852	0.863	0.784	0.757	0.082

Abbreviations: AUC, receiver operating characteristic curve; categorical boosting (CatBoost); SVM, support vector machine.

### 3.4 Model interpretation

**FIGURE 5:**
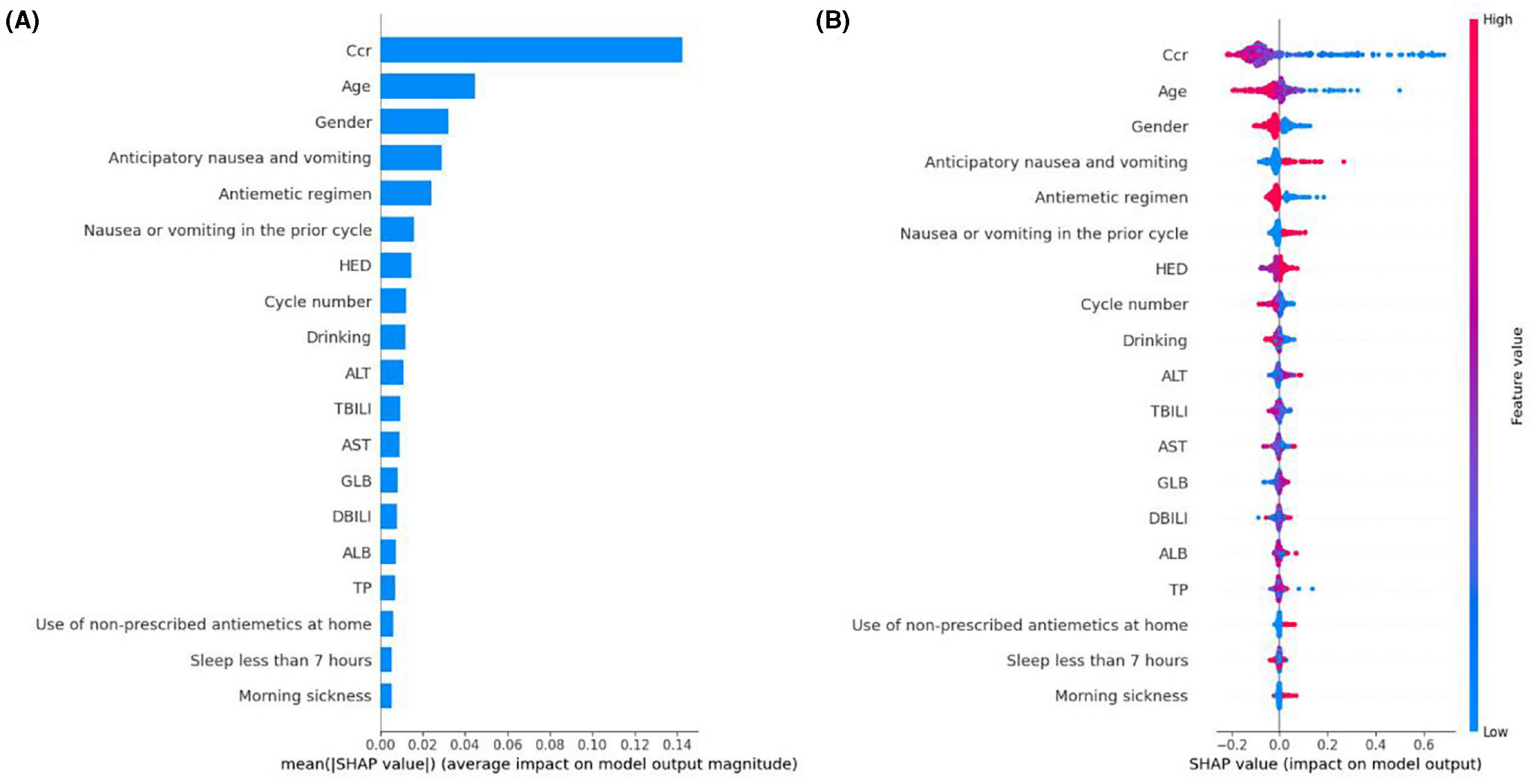
Matrix plots of features. (A), Bar plot of feature importance. (B), violin plot of SHAP summary. In the bar plot, the SHAP values represent the contribution of the particular features. A higher SHAP value indicates a more significant contribution of the model to a particular feature. Each dot represents an individual sample in the violin plot, and the color represents the value of each feature. The x‐axis represents the SHAP values, and a positive SHAP value indicates that it has positive effects on the predictions of the model, and vice versa. ALB, albumin; ALP, alkaline phosphatase; ALT, alanine transaminase; AST, aspartate transaminase; Ccr, creatinine clearance; DBILI, direct bilirubin; GLB, globulin; HED, highly emetogenic drug; TBILI, total bilirubin; TP, total proteins.

The SHAP summary of the deep forest models provided visualizations of the ranking of important features (Figure 5A) and the overall correlation and directionality between features and outcomes (Figure 5B). The top five most important features were Ccr, age, gender, anticipatory nausea and vomiting, and antiemetic regimen. Among these, Ccr played a critical role in predicting CINV. The SHAP summary indicated that lower Ccr values were associated with a higher risk of CINV. Anticipatory nausea and vomiting, female gender, and non‐standard antiemetic regimens were positively correlated with the risk of CINV. Additionally, lower age indicated a higher risk of CINV. The digital conversion of categorical variables is presented in Table S3. The correlation analysis revealed weak relationships between each variable (Figure S2).

**FIGURE 6:**
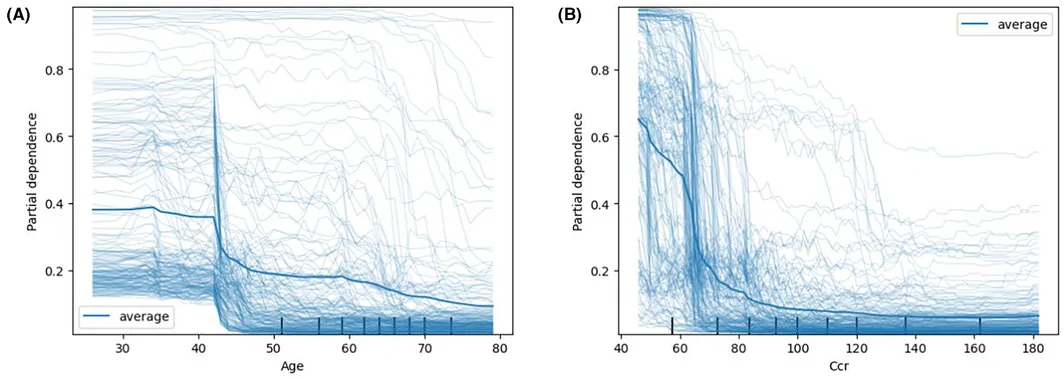
PDP of continuous variables in deep forest model. (A) Age; (B) Ccr. Ccr, creatinine clearance.

To further understand the impact of specific characteristics on the final prediction results, Individual Conditional Expectation (ICE) plots and PDP were employed. The ICE plot illustrates the trend of the prediction outcome with respect to each feature, while the PDP calculates the average effect of the features across all samples. The probability of CINV decreases with increasing age, and when the age exceeds 43 years, the probability of CINV decreases rapidly (Figure 6A). The probability of CINV increases with decreasing Ccr, and when Ccr falls below 70 mL/min, the probability of CINV rapidly increases (Figure 6B).

**FIGURE 7:**
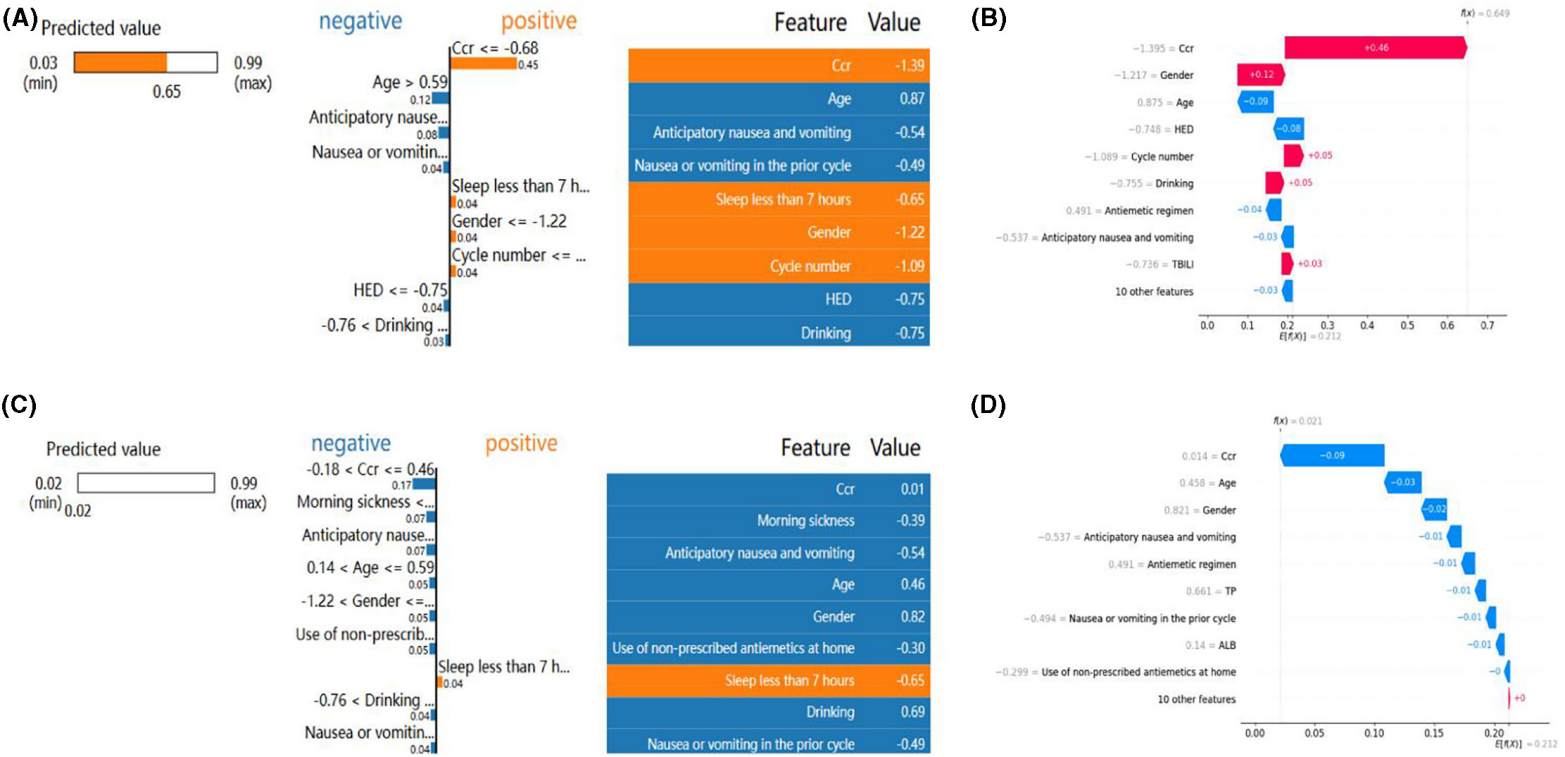
Specific prediction and interpretation of the deep forest model for two patients. (A,C) individual prediction interpretation based on the LIME method; (B,D) individual prediction interpretation based on SHAP.

Examples of applying the deep forest model for individual patient risk prediction are shown in Figure 7. The first patient had a predicted probability of developing CINV of 0.65. According to the explanations provided by both SHAP and LIME, lower Ccr and female gender after treatment were important factors contributing to the occurrence of CINV (Figure 7A,B). The second patient had a predicted probability of CINV of 0.02. Higher Ccr, older age, male gender, and the absence of anticipatory nausea and vomiting after treatment were the most significant determinants indicating a lower likelihood of developing CINV (Figure 7C,D).

## 4 DISCUSSION

### 4.1 Principal findings

Chemotherapy‐induced nausea and vomiting (CINV) remains a persistent challenge in the management of cancer patients undergoing chemotherapy. The ability to predict individual patient risk is crucial for guiding CINV management in the era of precision medicine. Several CINV risk assessment tools have been developed. Dranitsaris et al.^7^ established a tool for assessing acute and delayed phase CINV risk, achieving an AUC of 0.69, and incorporating psychological factors as variables for the first time. Zhi et al.^29^ constructed a nomogram prediction model for cancer patients receiving highly/moderately emetogenic chemotherapy, with a C‐index of 0.65 (95% CI, 0.58–0.72). However, these tools showed limited prediction ability, with AUC values below 0.7, whereas an AUC greater than 0.8 is generally considered acceptable. It is possible that previous studies did not identify all the risk factors. In our study, we included liver and kidney function as additional factors, based on previous risk factor studies, and this significantly improved the predictive performance of our model. Furthermore, Ccr emerged as the most influential factor in the model construction, further highlighting the importance of kidney function as a risk factor for CINV.

Among the top five important features in our study, Ccr, age, gender, anticipatory nausea and vomiting, and antiemetic regimen were identified. Ccr played the most significant role in CINV development, with the risk decreasing as Ccr increased, although the underlying mechanisms are not yet fully defined. This may be attributed to the fact that the majority of chemotherapy regimens administered to our patients included cisplatin or carboplatin, which are primarily eliminated through renal excretion. Thus, renal function can substantially impact their pharmacokinetics.^30^, ^31^, ^32^ A lower Ccr prolongs the presence of the drugs and their metabolites in the body, leading to continuous stimulation of the gastrointestinal tract, thereby increasing the risk of nausea and vomiting. Previous studies consistently identified females as being at higher risk for CINV,^6^, ^7^ which aligns with our findings in cancer patients prescribed highly emetogenic chemotherapy. Age and anticipatory nausea and vomiting have also been consistently reported as risk factors for CINV.^33^, ^34^, ^35^ Consistent with these findings, our study revealed that younger patients and those who expected nausea and vomiting had a higher risk of CINV. It has been shown that guideline‐recommended antiemetic regimens can prevent 65% of nausea and 85% of vomiting,^36^, ^37^ and our results support the use of guideline‐compliant CINV prophylaxis to reduce CINV incidence after highly emetogenic chemotherapy.

The deep forest model outperforms typical machine learning models in terms of predictive performance. This novel deep learning algorithm possesses robust computational power, assigning different weights to features based on their importance in predicting outcomes.^20^ With the accumulation of samples and the refinement of various medical indicators, the model is expandable and the selection of features is not necessary. To address the inherent complexity of deep learning models, we employed a combination of two interpretation techniques, SHAP and PDP, at multiple levels of the model (global, feature, and individual). This approach ensures stability and objectivity in result interpretation. Imbalanced data can introduce bias in the classifier towards the majority class, thus affecting the classification performance.^27^, ^28^ Generative adversarial networks (GANs) are an advanced deep generative algorithm that offers flexibility in model design and parallel generation, presenting notable advantages over other generative algorithms.^38^ However, GANs are not well‐suited for handling categorical data and often encounter training instability, leading to model collapse.^39^ On the other hand, SMOTE oversampling, based on the principle of similarity between neighboring points in the feature space, generates synthetic samples for minority classes using linear interpolation. It offers stability and is suitable for handling both categorical and continuous data.^40^ Given that our dataset comprised both categorical and continuous variables, SMOTE oversampling was the appropriate method to balance the data and reduce overfitting. In our study, we employed SMOTE oversampling and the deep forest model to develop a prediction model for personalized estimation of CINV development in cancer patients prescribed highly emetogenic chemotherapy (HEC), aiming to identify high‐risk patients. Furthermore, our team has developed software based on this model, enabling the generation of individualized CINV risk estimates prior to each chemotherapy cycle. Clinicians can input patient‐specific risk factors into the software to obtain the patient's risk of developing CINV, enabling them to tailor antiemetic interventions for high‐risk patients and prevent unnecessary nausea and vomiting.

### 4.2 Limitations and further work

While our prediction model offers potential benefits, there are several limitations to consider. Firstly, although our sample size comprised over 1700 chemotherapy cycles, the model lacks external validation. Additionally, the study population consisted solely of Chinese patients, warranting further verification of the model's applicability to other ethnic groups. Evidence suggests that Asian women may experience a higher incidence of CINV compared to non‐Asians.^41^ Furthermore, considering the distinct development mechanisms and influencing factors at different phases of CINV (acute, delayed, and extended), it is important to develop phase‐specific prediction models in the future to enhance the accuracy of CINV prediction.

## 5 CONCLUSIONS

In conclusion, we have developed an interpretable deep forest model that effectively identifies high‐risk individuals for CINV prediction in cancer patients undergoing HEC. This model outperforms typical machine learning models and incorporates liver and kidney function in conjunction with known individual variables and expands the scope of study endpoints. By enabling physicians to comprehensively assess individual CINV risk, this model facilitates informed decisions regarding antiemetic interventions for cancer patients receiving HEC treatment.

## AUTHOR CONTRIBUTIONS

**Jingyue Zhang:** Formal analysis (equal); methodology (equal); writing – original draft (lead). **Xudong Cui:** Software (equal); visualization (equal); writing – original draft (equal). **Chong Yang:** Data curation (equal); investigation (equal). **Diansheng Zhong:** Conceptualization (equal); data curation (equal). **Yinjuan Sun:** Data curation (equal). **Xiaoxiong Yue:** Software (equal); visualization (equal). **Gaoshaung Lan:** Data curation (equal). **Linlin Zhang:** Conceptualization (equal); data curation (equal); project administration (equal). **Liangfu Lu:** Formal analysis (lead); methodology (lead); software (lead). **hengjie Yuan:** Conceptualization (lead); project administration (lead); resources (lead); supervision (lead).

## FUNDING INFORMATION

Funding for this study was provided by the National Natural Science Foundation of China (grant number: 81501057, 81,971,173, 81,102,447) and the Tianjin Municipal Science and Technology Commission (grant number: 16JCQNJC10600).

## CONFLICT OF INTEREST STATEMENT

The authors declare no conflict of interest.

## Supporting information


Data S1.

## Data Availability

All data are available from the corresponding author. And the complete source code is available on https://github.com/Xgll6/Machine‐learning.
