# Supplementary material for: A deep learning‐based interpretable decision tool for predicting high risk of chemotherapy‐induced nausea and vomiting in cancer patients prescribed highly emetogenic chemotherapy
Source: Cancer Med. 2023 Aug 23;12(17):18306–16. doi: 10.1002/cam4.6428 (PMC10524079; doi:10.1002/cam4.6428)
Supplement: Supplementary file 1 — Data S1. [file CAM4-12-18306-s001.pdf]

Supplemental Material

A Deep Learning-Based Interpretable Decision Tool for Predicting High Risk of Chemotherapy-Induced Nausea and Vomiting in Cancer Patients Prescribed Highly Emetogenic Chemotherapy

Supplemental Tables

I. Missing values

II. Hyperparameter search domains and final settings of six model

III. Digital conversion of categorial variables

Supplemental Figures

I. Overview of the prediction model framework based on deep forest.

II. Correlation between variables.

Supplemental Tables

Table I. Missing values

Variables	Variable type	Missing values N (%)
History of drinking	Categorial	679(38.8%)
Cycle number	Categorial	3(0.2%)
TP	Continuous	123(7.0%)
ALB	Continuous	109(6.2%)
GLB	Continuous	129(7.4%)
AST	Continuous	105(6.0%)
ALT	Continuous	105(6.0%)
TBILI	Continuous	115(6.6%)
DBILI	Continuous	261(14.9%)
ALP	Continuous	123(7.0%)

Abbreviations; TP, total proteins; ALB, albumin; GLB, globulin; AST, aspartate transaminase; ALT, alanine transaminase; TBILI, total bilirubin; DBILI, direct bilirubin; ALP, alkaline phosphatase.

Table II. Hyperparameter search domains and final settings of six model

Hyperparameter	Final setting
**Neural Network**	
'units'	23
'batch_size'	20
**CatBoost**	
'loss_function'	MultiRMSE
'iterations'	119
'learning_rate'	0.018191183503577492
'depth'	9
'bagging_temperature'	0.02168908380387344
**Decision Tree**	
'criterion'	friedman_mse
'max_depth'	12
**SVM**	
'kernel'	rbf
**Random Forest**	
'criterion'	absolute_error
'n_estimators'	88
'min_samples_split'	9
'min_samples_leaf'	3
**Deep Forest**	
'max_layers'	3
'n_estimators'	10

'min_samples_split'	3
'min_samples_leaf'	2
'delta'	3.508e-06

Abbreviations; SVM, support vector machine; CatBoost, categorical boosting.

Table III. Digital conversion of categorial variables

**Categorial variables**	**Digital conversion**
Anticipatory nausea and vomiting	0-No, 1-Yes
Antiemetic regimen	0-standard antiemetic regimen; 1-non-standard antiemetic regimen
HED	0-AC based, 1-AC and cisplatin based, 2-Carboplatin AUC ⩾ 4 based, 3-Cyclophosphamide based, 4-Anthracycline based, 5-Anthracycline and cisplatin based, 6-Cisplatin based
Dringking	0-No, 1-Yes
Nausea or vomiting in the prior cycle	0-No, 1-Yes
Gender	0-Female, 1-Male
Sleep < 7 h before chemotherapy	0-No, 1-Yes
Morning sickness	0-No, 1-Yes
Use of non-prescribed antiemetics at home	0-No, 1-Yes

Abbreviations; HED, highly emetogenic drug; AC, Anthracycline and cyclophosphamide.

Supplemental Figures

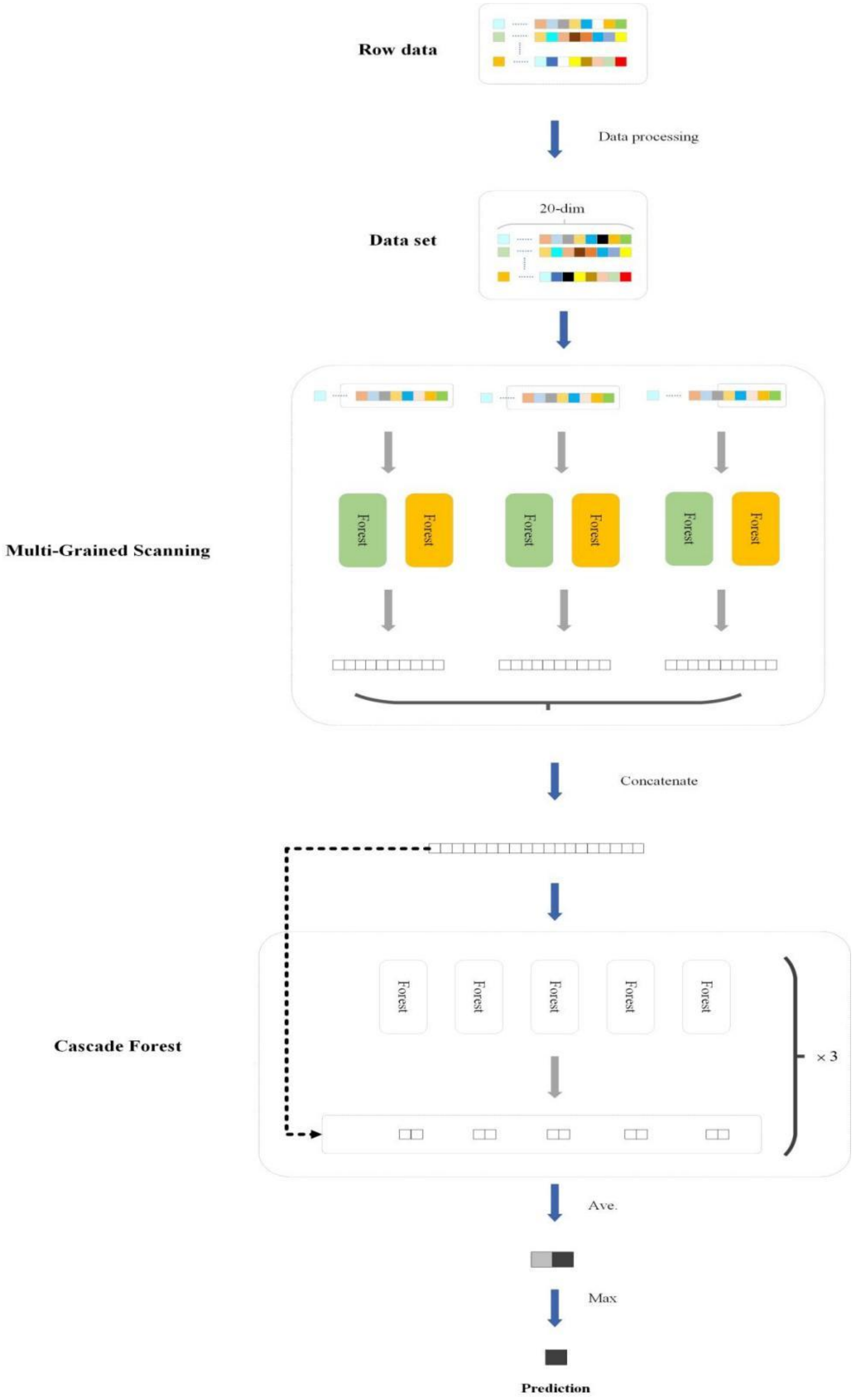

Figure I. Overview of the prediction model framework based on deep forest.

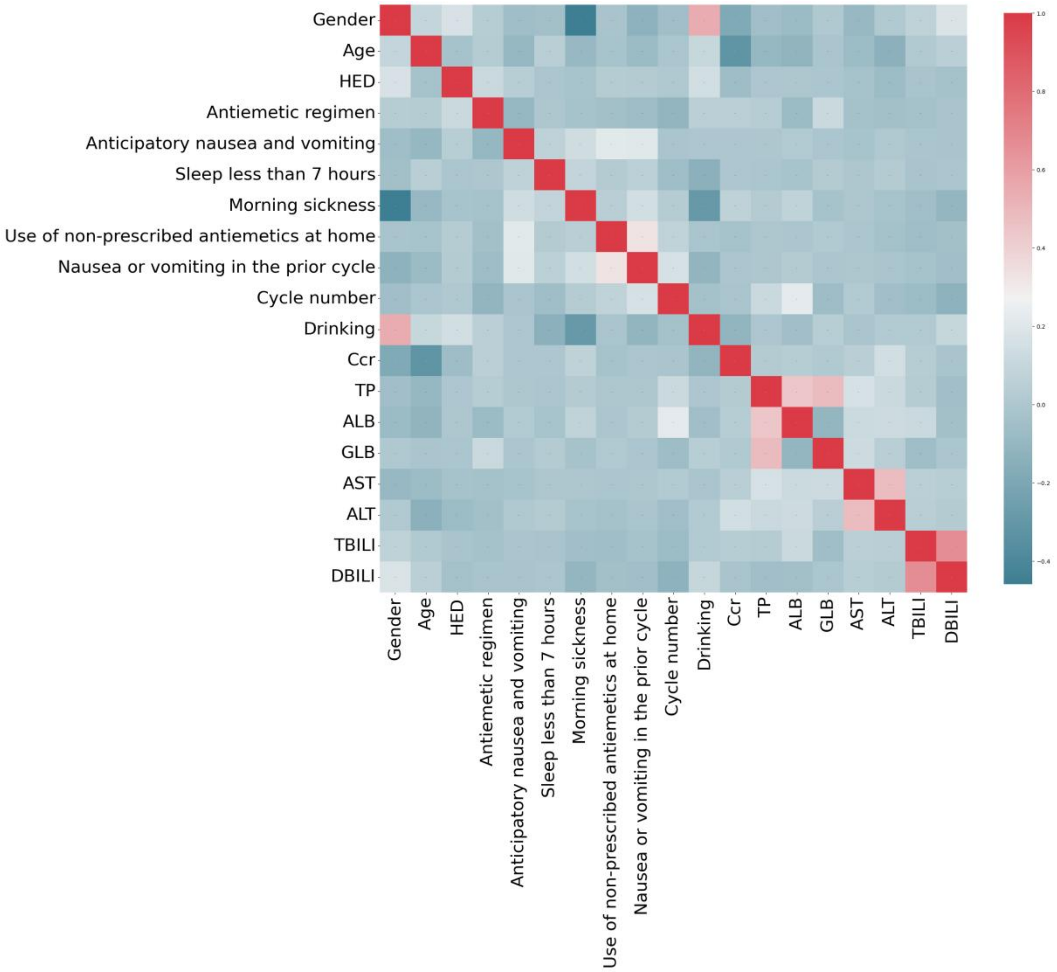

Figure II. Correlation between variables.
